# Autophosphorylation of Ser-6 via an intermolecular mechanism is important for the rapid reduction of NtCDPK1 kinase activity for substrate RSG

**DOI:** 10.1371/journal.pone.0196357

**Published:** 2018-04-23

**Authors:** Takeshi Ito, Sarahmi Ishida, Yohsuke Takahashi

**Affiliations:** 1 Department of Biological Science, Graduate School of Science, Hiroshima University, Higashi-Hiroshima, Japan; 2 Department of Biological Sciences, Graduate School of Science, University of Tokyo, Tokyo, Japan; National Taiwan University, TAIWAN

## Abstract

Tobacco (*Nicotiana tabacum*) Ca^2+^-dependent protein kinase 1 (NtCDPK1) is involved in feedback regulation of the plant hormone gibberellin through the phosphorylation of the transcription factor, REPRESSION OF SHOOT GROWTH (RSG). Previously, Ser-6 and Thr-21 were identified as autophosphorylation sites in NtCDPK1. Autophosphorylation of Ser-6 and Thr-21 not only decreases the binding affinity of NtCDPK1 for RSG, but also inhibits the homodimerization of NtCDPK1. Furthermore, autophosphorylation decreases the phosphorylation efficiency of RSG. We demonstrated that Ser-6 and Thr-21 of NtCDPK1 are phosphorylated in response to GAs in plants. The substitution of these autophosphorylation sites with Ala enhances the NtCDPK1 overexpression-induced sensitization of seeds to a GA biosynthetic inhibitor during germination. These findings suggested that autophosphorylation of Ser-6 and Thr-21 prevents excessive phosphorylation of RSG. In this study, we attempted to determine which autophosphorylation site is responsible for the functional regulation of NtCDPK1. Ser-6 was autophosphorylated within 1 min, whereas Thr-21 required over 5 min to be completely autophosphorylated. Furthermore, we found that Ser-6 and Thr-21 were autophosphorylated by inter- and intramolecular mechanisms, respectively, which may be reflected in the faster autophosphorylation of Ser-6. Although both autophosphorylation sites were involved in the reduction of the binding affinity of NtCDPK1 for RSG and the inhibition of NtCDPK1 homodimerization, autophosphorylation of Ser-6 alone was sufficient to decrease the kinase activity of NtCDPK1 for RSG. These results suggest that autophosphorylation of Ser-6 is important for the rapid reduction of NtCDPK1 kinase activity for RSG, whereas that of Thr-21 may play an auxiliary role.

## Introduction

Ca^2+^ is an important second messenger in plants and other eukaryotes [1]. Environmental information is converted into stimulus-specific temporal changes in cytosolic Ca^2+^ concentration ([Ca^2+^]_cyt_) that can vary in amplitude, frequency, duration, intracellular location, and timing [2]. Ca^2+^-dependent protein kinases (CDPKs) and calcineurin B-like-interacting protein kinases (CIPKs) are thought to play central roles in Ca^2+^ signaling in plants because protein kinase C and conventional Ca^2+^/calmodulin-dependent protein kinase (CaMK), which represent the two major types of Ca^2+^-regulated kinases in animals, are missing from *Arabidopsis thaliana* [3,4]. CDPKs are only found in plants and some protists including malarial parasites [5] and play important roles in various physiological processes including growth, development, and responses to biotic and abiotic stresses in plants [6–8]. CDPKs are multifunctional Ser/Thr protein kinases possessing both Ca^2+^-sensing and signaling capabilities within a single gene product [9,10] and composed of a variable N-terminal domain, a catalytic domain, a junction domain containing an autoinhibitory segment, and a calmodulin (CaM)-like domain [11,12]. The variable N-terminal domains of many CDPKs are predicted to be involved in the association of CDPKs with the plasma membrane by being myristoylated and palmitoylated [3,6,8]. The variable N-terminal domain is also involved in substrate recognition [13,14], while the autoinhibitory segment blocks the substrate binding site of the catalytic domain in the absence of Ca^2+^. Ca^2+^ binding to the CaM-like domain triggers the translocation of the junction domain and CaM-like domain, exposing the residues required for kinase activity and substrate recognition [15,16].

Tobacco (*Nicotiana tabacum*) NtCDPK1 was identified as a protein kinase that phosphorylates REPRESSION OF SHOOT GROWTH (RSG) [17]. RSG is a basic leucine zipper transcriptional activator, which regulates the transcription of gibberellin (GA) biosynthetic genes [18,19]. Binding of 14-3-3 proteins to RSG is dependent on phosphorylation of RSG residue Ser-114, which sequesters RSG in the cytoplasm, resulting in reduced expression of target genes [20,21]. GA levels regulate the intracellular localization of RSG, which is translocated into the nucleus in response to a reduction in GA levels, but GA treatment can reverse nuclear accumulation [21]. NtCDPK1 promotes 14-3-3 binding to RSG by phosphorylating Ser-114 [17]. In the presence of Ca^2+^, RSG and 14-3-3 form a heterotrimer with NtCDPK1 by binding to the variable N-terminal domain and the catalytic domain of NtCDPK1, respectively [22,23]. NtCDPK1 transfers 14-3-3 to RSG after phosphorylation of RSG, and RSG dissociates from NtCDPK1 as a complex with 14-3-3. Therefore, NtCDPK1 acts as a scaffolding kinase that increases the specificity and efficiency of signaling by coupling catalysis with scaffolding on the same protein.

Some kinases require specific upstream kinases for activation, but many kinases possess inherent autophosphorylation activity. Approximately 45% of all human protein kinases can reportedly autophosphorylate their activation loop [24]. Autophosphorylation of the activation loop in the catalytic domain is a fundamental and conserved mechanism of activation in many protein kinases [25–27]. For example, cAMP-dependent protein kinase A is activated by phosphorylation at Thr-197 in the activation loop [28]. However, the equivalent position in CDPKs is Asp or Glu, the negatively charged carboxylate of which mimics the phosphoactivated state, suggesting that CDPKs exhibit full activity without phosphorylation in the activation loop. Thus, the catalytic activity of CDPKs is exclusively regulated by the concentration of Ca^2+^. However, the functions of autophosphorylation outside the activation loop remain mostly unclear.

We recently revealed that NtCDPK1 can be autophosphorylated via an intermolecular mechanism, and Ser-6 and Thr-21 in the variable N-terminal domain were identified as autophosphorylation sites [29]. Autophosphorylation not only reduced the binding affinity of NtCDPK1 for RSG, but also inhibited homodimerization of NtCDPK1. Furthermore, autophosphorylation decreased the phosphorylation efficiency of RSG, but increased that of myelin basic protein as a conventional substrate. Ser-6 and Thr-21 of NtCDPK1 were phosphorylated in response to GAs in plants. NtCDPK1 is involved in the feedback regulation of the expression of a GA biosynthetic gene, *NtGA20ox1* [17]. Overexpression of NtCDPK1 causes the sensitization of seed germination to a GA biosynthetic inhibitor through the suppression of up-regulation of *NtGA20ox1* in transgenic plants. The substitution of Ser-6 and Thr-21 with Ala resulted in the reduction from 42.3% to 32.9% of the seed germination rate [29]. These results suggested that autophosphorylation can prevent excessive phosphorylation of RSG in plants. However, it was unclear whether autophosphorylation of Ser-6 and/or Thr-21 is involved in the regulation of NtCDPK1. In this study, we aimed to determine which autophosphorylation site is responsible for the functional regulation of NtCDPK1.

## Materials and methods

### Preparation of recombinant proteins

Plasmids pET16b-NtCDPK1 wild-type (WT), pGEX-4T-1-NtCDPK1 WT, S6A, T21A, S6A/T21A, D219N, and S6A/T21A/D219N, and pMALc2-RSG were previously generated [17,29]. NtCDPK1 S6A/D219N and T21A/D219N were generated using overlap extension PCR and seamless ligation cloning extract (SLiCE) [30] with a plasmid harbouring full-length NtCDPK1 as a template and appropriate primers (S1 Table). PCR products were cloned into pGEX-4T-1 (GE Healthcare). Glutathione *S*-transferase (GST)-NtCDPK1s, maltose-binding protein (MBP)-RSG, and 10×His-tagged (His)-NtCDPK1 were expressed in *Escherichia coli* harbouring pGEX-4T-1-NtCDPK1, pMALc2-RSG, or pET16b-NtCDPK1, respectively, and purified by glutathione-Sepharose 4B (GE Healthcare), Amylose Resin (New England Biolabs), or COSMOGEL His-Accept (Nacalai Tesque), respectively.

### Autophosphorylation reaction

GST-NtCDPK1 proteins (2.0 μg) were reacted in autophosphorylation buffer containing 20 mM Tris-HCl, pH 7.5, 10 mM MgCl_2_, 0.5 mM CaCl_2_, 0.1% (v/v) Triton X-100, 0.05% (v/v) β-mercaptoethanol, and 1 mM ATP at 30°C for 1 h. Catalytically inactive forms of GST-NtCDPK1, GST-NtCDPK1 D219N, GST-NtCDPK1 S6A/D219N, GST-NtCDPK1 T21A/D219N and GST-NtCDPK1 S6A/T21A/D219N, were autophosphorylated by His-NtCDPK1 in autophosphorylation buffer at 30°C for 1 h. The phosphorylation state was examined using Phosphate-binding tag (Phos-tag) SDS-PAGE [31].

### *In vitro* pull-down assay

GST-NtCDPK1 (2.0 μg) was incubated with 20 μL glutathione-Sepharose 4B (GE Healthcare) and MBP-RSG (3.0 μg) or His-NtCDPK1 (3.0 μg) in 400 μL of binding buffer containing 25 mM MOPS-NaOH, pH 7.5, 25 mM NaCl, 0.05% (v/v) β-mercaptoethanol, 0.1% (v/v) Triton X-100, 0.1 mM phenylmethylsulfonyl fluoride, and 0.5 mM CaCl_2_ at 4°C for 30 min. Proteins bound to beads were washed with binding buffer, resolved by SDS-PAGE (Tris/glycine buffer) or Phos-tag SDS-PAGE, and detected by Coomassie Brilliant Blue (CBB) staining or immunoblotting as described below.

### *In vitro* kinase assay

The catalytic activity of GST-NtCDPK1 proteins was assayed in autophosphorylation buffer as described above at 30°C. GST-NtCDPK1 (1.0 μg/mL) and MBP-RSG (0.2 mg/mL) were used for the assay. After aliquots of reaction mixture were subjected to SDS-PAGE, phosphorylated RSG was detected by immunoblotting as described below. The intensities of bands were measured using ImageJ software (NIH, version 1.50i).

### Immunoblotting analysis

After electrophoresis, separated proteins were transferred onto an Immobilon-P transfer membrane (Millipore), and a wet transfer method was used for Phos-tag SDS-PAGE [32]. Membranes were probed with anti-NtCDPK1 [17], anti-GST (GE Healthcare), anti-MBP (MBL), or anti-pS114 (antibody against the phosphorylated Ser-114 of RSG) [21], followed by horseradish peroxidase-conjugated secondary antibody. Chemiluminescence was detected using Immobilon Western Chemiluminescent HRP Substrate (Millipore) and quantified using ImageQuant LAS 4000 with ImageQuant TL software (GE Healthcare).

### Statistical analysis

Statistical analysis was performed using R (version 3.2.3, https://www.r-project.org/). Comparisons were performed using one-way ANOVA with Tukey’s honestly significant difference test.

### Accession numbers

Sequence data from this article can be found in GenBank/EMBL data libraries under accession numbers AF072908 (NtCDPK1) and AB040471 (RSG).

## Results

### Ser-6 is autophosphorylated faster than Thr-21

**Fig 1:**
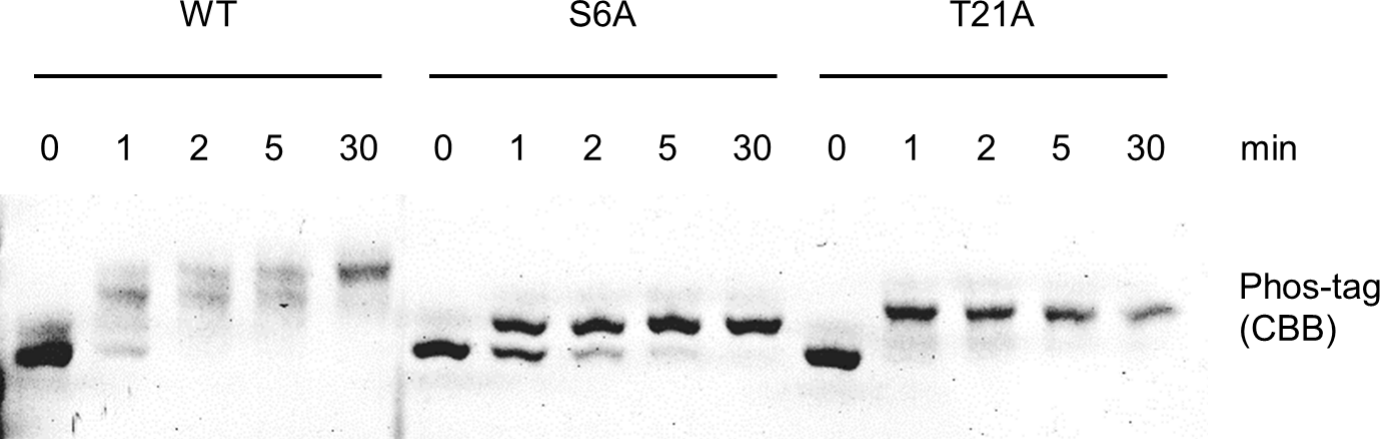
Ser-6 of NtCDPK1 is autophosphorylated faster than Thr-21. GST-NtCDPK1 WT, GST-NtCDPK1 S6A, and GST-NtCDPK1 T21A were autophosphorylated for the indicated time periods. The autophosphorylation state of GST-NtCDPK1 proteins was examined using Phos-tag SDS-PAGE. GST-NtCDPK1 proteins were visualized by Coomassie Brilliant Blue (CBB) staining. This experiment was repeated three times with similar results.

Autophosphorylation of Ser-6 and Thr-21 decreases the kinase activity of NtCDPK1 for RSG within 2 min of starting the reaction [29], indicating rapid autophosphorylation. In phosphate-binding tag (Phos-tag) SDS-PAGE, phosphorylated protein bands migrate more slowly [33]. To examine whether NtCDPK1 is already autophosphorylated within 2 min, we determined the autophosphorylation level of glutathione *S*-transferase (GST)-fused NtCDPK1 proteins using Phos-tag SDS-PAGE. Multiple bands of autophosphorylated GST-NtCDPK1 wild-type (WT) were observed at 1, 2, and 5 min, and a single band was observed at 30 min (Fig 1). This result suggests that either Ser-6 or Thr-21 is autophosphorylated within 1–5 min, and the autophosphorylation rate differs between Ser-6 and Thr-21. To examine the autophosphorylation rates of Ser-6 and Thr-21, we used GST-NtCDPK1 S6A and GST-NtCDPK1 T21A, in which Ser-6 and Thr-21 were substituted with Ala, respectively [29]. Phos-tag SDS-PAGE showed that GST-NtCDPK1 T21A was autophosphorylated within 1 min, whereas GST-NtCDPK1 S6A required over 5 min to be completely autophosphorylated (Fig 1). These results suggest that Ser-6 is autophosphorylated faster than Thr-21.

### Ser-6 and Thr-21 are autophosphorylated by different mechanisms

**Fig 2:**
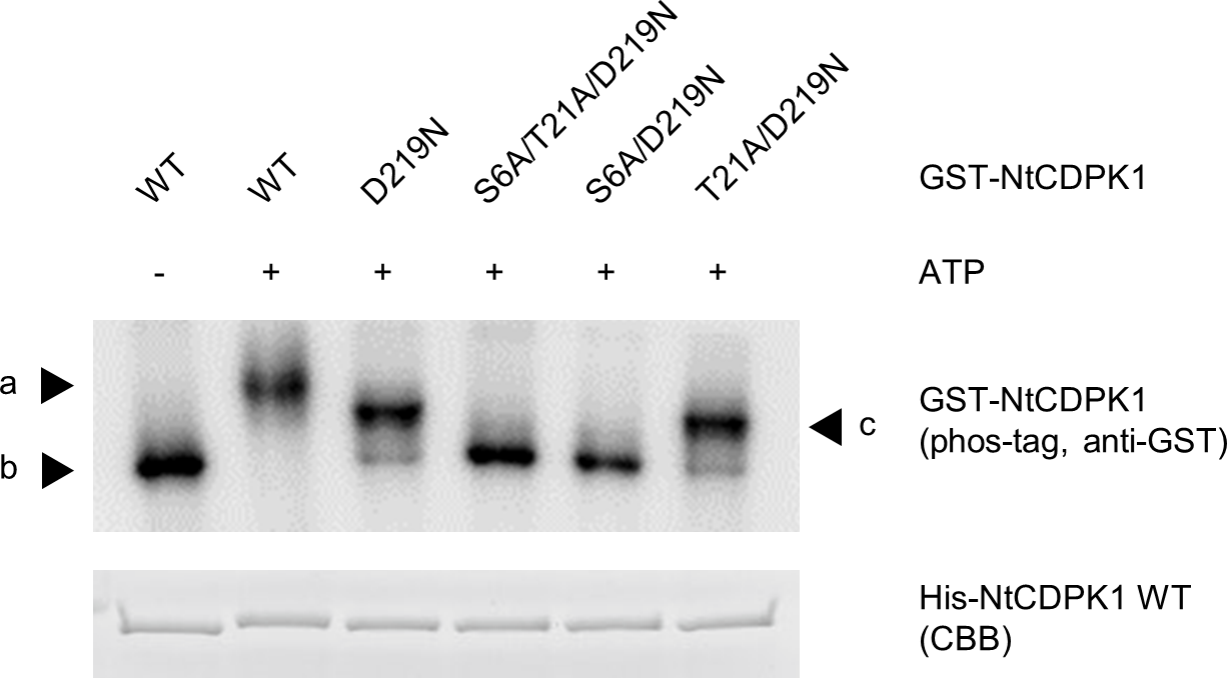
Ser-6 and Thr-21 are autophosphorylated by different mechanisms. Phosphorylation of GST-NtCDPK1 proteins by His-NtCDPK1 was by reacting GST-NtCDPK1 WT, GST-NtCDPK1 D219N, GST-NtCDPK1 S6A/D219N, GST-NtCDPK1 T21A/D219N, and GST-NtCDPK1 S6A/T21A/D219N with His-NtCDPK1 WT in autophosphorylation buffer. The autophosphorylation state of GST-NtCDPK1 proteins was examined using Phos-tag SDS-PAGE. GST-NtCDPK1 proteins were detected by immunoblotting with anti-GST antibody. His-NtCDPK1 WT was visualized by CBB staining. Letters represent phosphorylated GST-NtCDPK1 WT (a), unphosphorylated GST-NtCDPK1 WT, GST-NtCDPK1 S6A/D219N, and GST-NtCDPK1 S6A/T21A/D219N (b), and phosphorylated GST-NtCDPK1 D219N and GST-NtCDPK1 T21A/D219N (c). This experiment was repeated three times with similar results.

We previously found that NtCDPK1 can be autophosphorylated via an intermolecular mechanism [29]. However, because the autophosphorylation rate differs between Ser-6 and Thr-21 (Fig 1), we assumed that the autophosphorylation mechanism may differ between Ser-6 and Thr-21. A variant of NtCDPK1 with Asp-219 replaced by Asn is catalytically inactive and is not intramolecularly autophosphorylated because Asp-219 of NtCDPK1 is an essential residue for catalytic activity [29,34,35]. We constructed catalytically inactive forms of NtCDPK1, GST-NtCDPK1 D219N, GST-NtCDPK1 S6A/D219N, GST-NtCDPK1 T21A/D219N, and GST-NtCDPK1 S6A/T21A/D219N to examine whether Ser-6 and/or Thr-21 of GST-NtCDPK1 are trans-phosphorylated by 10×His-tagged (His)-NtCDPK1 (Fig 2). Phos-tag SDS-PAGE showed that GST-NtCDPK1 T21A/D219N migrated slower than S6A/D219N, and migration of GST-NtCDPK1 S6A/D219N was comparable to that of unphosphorylated GST-NtCDPK1 WT and GST-NtCDPK1 S6A/T21A/D219N, suggesting that Thr-21 is not trans-phosphorylated by His-NtCDPK1. Meanwhile, migration of GST-NtCDPK1 T21A/D219N was comparable to that of GST-NtCDPK1 D219N, suggesting that Ser-6 was trans-phosphorylated by His-NtCDPK1. Thus, Ser-6 can be autophosphorylated in an intermolecular manner, whereas Thr-21 cannot. Migration of GST-NtCDPK1 WT was even slower than that of GST-NtCDPK1 D219N and GST-NtCDPK1 T21A/D219N, suggesting that Ser-6 and Thr-21 in GST-NtCDPK1 WT are autophosphorylated by inter- and intramolecular mechanisms, respectively. Furthermore, these results indicate that the faster autophosphorylation of Ser-6, as shown in Fig 1, may reflect the difference in autophosphorylation mechanisms.

### NtCDPK1 homodimerization is inhibited when either Ser-6 or Thr-21 is autophosphorylated

**Fig 3:**
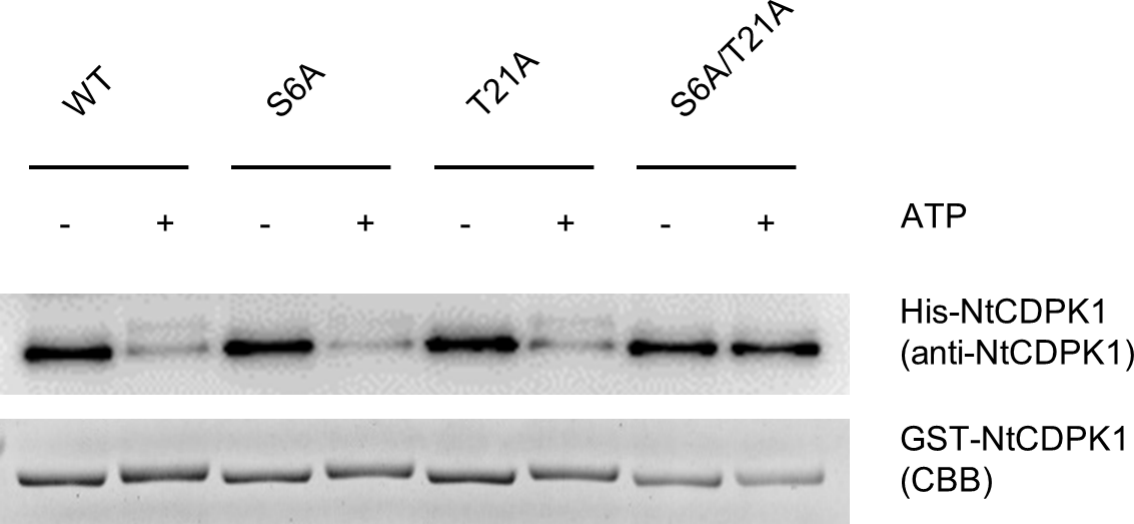
NtCDPK1 homodimerization is decreased by autophosphorylation of either Ser-6 or Thr-21. Autophosphorylated and unphosphorylated GST-NtCDPK1 were immobilized on glutathione beads. After washing, GST-NtCDPK1-immobilized beads were incubated with His-NtCDPK1. GST-NtCDPK1-bound proteins were subjected to SDS-PAGE, followed by immunoblotting with anti-NtCDPK1 antibody for the detection of His-NtCDPK1. GST-NtCDPK1 proteins were visualized by CBB staining. This experiment was repeated three times with similar results.

NtCDPK1 forms a homodimer in a Ca^2+^-dependent manner [29]. Autophosphorylation decreases not only the binding of NtCDPK1 to RSG, but also the dimerization of NtCDPK1. To examine the effect of individual autophosphorylation of Ser-6 and Thr-21 on dimerization of NtCDPK1, an *in vitro* pull-down assay was performed using GST-NtCDPK1 and His-NtCDPK1. Autophosphorylated and unphosphorylated GST-NtCDPK1 WT, GST-NtCDPK1 S6A, GST-NtCDPK1 T21A, and GST-NtCDPK1 S6A/T21A were immobilized on glutathione beads and incubated with His-NtCDPK1 WT (Fig 3). The results showed that binding of His-NtCDPK1 to GST-NtCDPK1 WT, GST-NtCDPK1 S6A, and GST-NtCDPK1 T21A was decreased by autophosphorylation, but binding of GST-NtCDPK1 S6A/T21A was not. This result suggests that homodimerization of NtCDPK1 is inhibited when either Ser-6 or Thr-21 is autophosphorylated.

### NtCDPK1 decreases the binding affinity for RSG when either Ser-6 or Thr-21 is autophosphorylated

**Fig 4:**
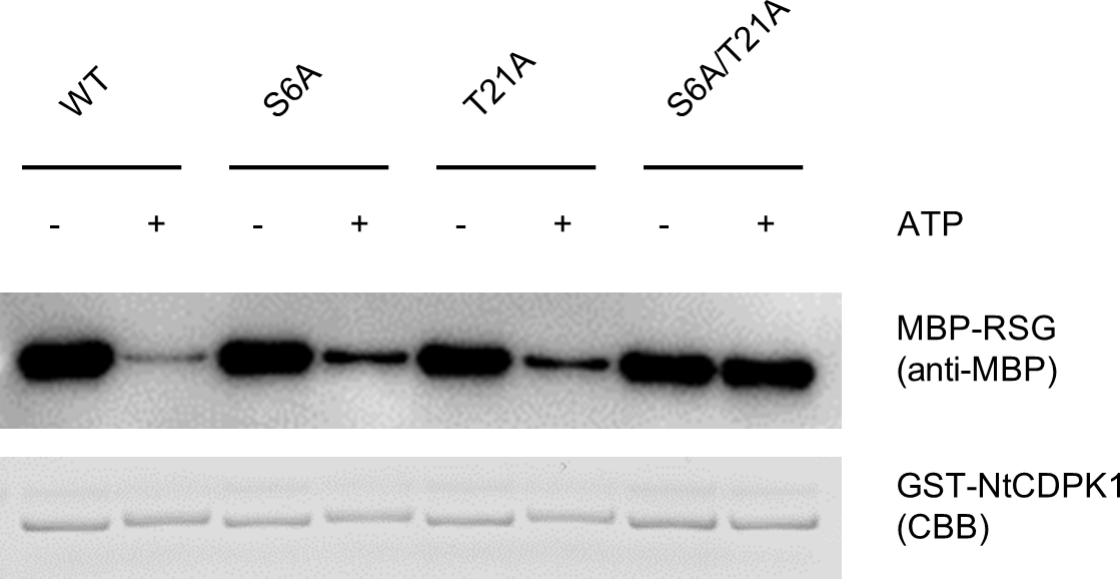
The binding affinity of NtCDPK1 for RSG is decreased by autophosphorylation of either Ser-6 or Thr-21. Autophosphorylated and unphosphorylated GST-NtCDPK1 were immobilized on glutathione beads. After washing, GST-NtCDPK1-immobilized beads were incubated with MBP-RSG. GST-NtCDPK1-bound proteins were subjected to SDS-PAGE, followed by immunoblotting with anti-MBP antibody for the detection of MBP-RSG. GST-NtCDPK1 proteins were visualized by CBB staining. This experiment was repeated three times with similar results.

We next examined the effect of individual autophosphorylation of Ser-6 and Thr-21 on the binding affinity of NtCDPK1 for RSG using an *in vitro* pull-down assay because a previous study showed that binding of NtCDPK1 to RSG is decreased following autophosphorylation [29]. Autophosphorylated and unphosphorylated GST-NtCDPK1 proteins were immobilized on glutathione beads and incubated with maltose-binding protein (MBP)-RSG (Fig 4). When GST-NtCDPK1 was reacted in autophosphorylation buffer, GST-NtCDPK1 S6A and GST-NtCDPK1 T21A decreased the binding to MBP-RSG as well as GST-NtCDPK1 WT, but GST-NtCDPK1 S6A/ T21A did not. This result suggests that NtCDPK1 decreases the binding affinity for RSG when either Ser-6 or Thr-21 is autophosphorylated.

### Autophosphorylation of Ser-6 rapidly decreases NtCDPK1 kinase activity for RSG

**Fig 5:**
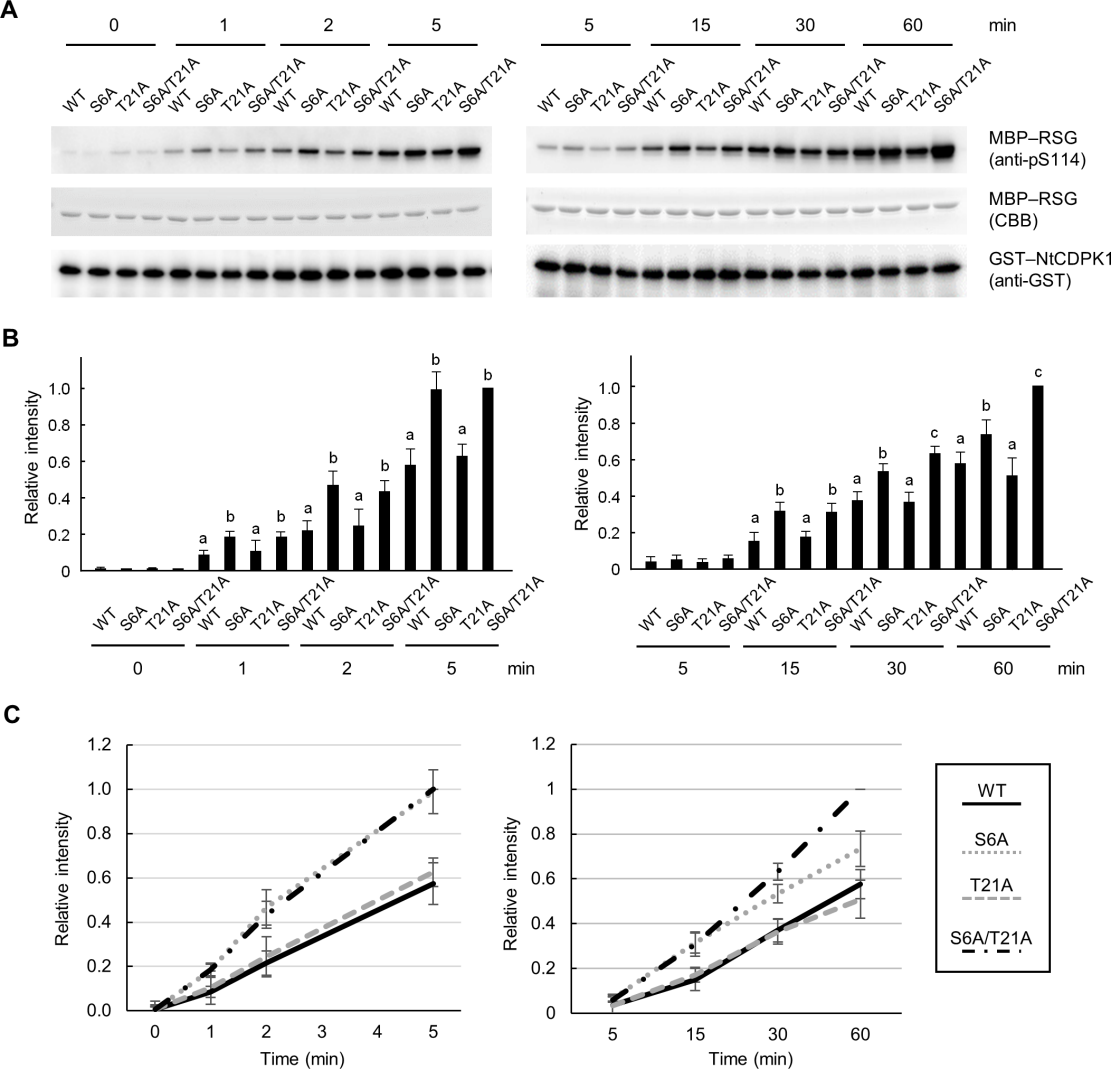
Autophosphorylation of Ser-6 and Thr-21 leads to rapid and slow inhibition of phosphorylation of RSG, respectively. (A) MBP-RSG was phosphorylated for the indicated time periods. Phosphorylation of Ser-114 of RSG was detected by immunoblotting using anti-pS114 antibody, which specifically recognizes phosphorylated Ser-114 of RSG. MBP-RSG was visualized by CBB staining. GST-NtCDPK1 proteins were detected by immunoblotting with anti-GST antibody. This experiment was repeated three times with similar results. (B) Statistical analysis of the data shown in (A). The quantified band intensity of phosphorylated RSG by GST-NtCDPK1 S6A/T21A at 5 min (left panel) and 60 min (right panel) was set to 1, respectively. The bar graph represents means and ± SE (*n* = 3). Significant differences in the phosphorylation of MBP-RSG by GST-NtCDPK1 proteins are determined by one-way ANOVA with Tukey’s honestly significant difference test at each time point. Different letters above the bars indicate significant differences between the relative intensities at each time point (*P* < 0.05). (C) Line graph represents the band intensities of phosphorylated RSG obtained from (A) and (B).

Autophosphorylation of NtCDPK1 negatively regulates the phosphorylation of RSG by decreasing the binding affinity [29]. Because the binding affinity of NtCDPK1 for RSG was decreased by autophosphorylation of either Ser-6 or Thr-21 (Fig 4), and because Ser-6 is autophosphorylated faster than Thr-21 (Fig 1), we predicted that autophosphorylation of Ser-6 and Thr-21 is responsible for the rapid and slow reduction in kinase activity toward RSG, respectively. To test this, we compared the time course of kinase activity between GST-NtCDPK1 WT, GST-NtCDPK1 S6A, GST-NtCDPK1 T21A, and GST-NtCDPK1 S6A/T21A using MBP-RSG as a substrate. At all timepoints, the kinase activity of NtCDPK1 T21A for RSG was comparable with that of NtCDPK1 WT (Fig 5). Phosphorylation of MBP-RSG was increased in the S6A or S6A/T21A mutants at 1–15 min, but NtCDPK1 S6A did not phosphorylate MBP-RSG as efficiently as NtCDPK1 S6A/T21A at 30 and 60 min. These results suggest that autophosphorylation of Ser-6 alone is sufficient to rapidly decrease the kinase activity of NtCDPK1 for RSG, and autophosphorylation of Thr-21 may play an auxiliary role in decreasing NtCDPK1 kinase activity for RSG.

## Discussion

*In vitro* autophosphorylation sites have been mapped for several CDPKs [36,37]. However, little is known about the autophosphorylation mechanism of CDPKs. NtCDPK2 participates in plant defence signaling, and Thr-65 in the variable N-terminal domain is autophosphorylated in response to stress [38]. The catalytically inactive form of NtCDPK2 (D241A mutant) is not trans-phosphorylated, suggesting that Thr-65 is autophosphorylated in an intramolecular manner. In the present study, we revealed that Ser-6 and Thr-21 of NtCDPK1 are autophosphorylated in an inter- and intramolecular manner, respectively (Fig 2). To our knowledge, this is the first report of a CDPK containing two sites that are autophosphorylated by different mechanisms. However, we cannot exclude the possibility that Ser-6 is not autophosphorylated in an intramolecular manner at present.

In this study, we revealed that autophosphorylation of Ser-6 by an intermolecular mechanism occurs faster than autophosphorylation of Thr-21 by an intramolecular mechanism (Figs 1 and 2). This finding raises questions about why only Ser-6 is autophosphorylated in an intermolecular manner in spite of the close proximity of Ser-6 and Thr-21, and why autophosphorylation by an intermolecular mechanism is faster than by an intramolecular mechanism. Protein interactions often regulate kinase activity through allosteric effects [39,40]. Dimerization of NtCDPK1 might contribute to stabilization of a unique conformation that promotes autophosphorylation of Ser-6 by an intermolecular mechanism. For example, dimerization might help Ser-6 of one NtCDPK1 monomer to orient the active site of another NtCDPK1 monomer, allowing Ser-6 to be rapidly autophosphorylated by an intermolecular mechanism. Furthermore, Thr-21 might be structurally unlikely to access the active site of another NtCDPK1 monomer despite being positioned close to Ser-6. In the presence of Ca^2+^, NtCDPK1 forms a homodimer and is immediately autophosphorylated, which causes the dissociation of the NtCDPK1 homodimer. Therefore, NtCDPK1 is believed to form a transient homodimer, but dimerization may be required for the efficient phosphorylation of Ser-6 via an intermolecular mechanism. A mutant version of NtCDPK1 that does not form a homodimer could verify the role of dimerization in the intermolecular autophosphorylation of Ser-6.

Our results suggest that autophosphorylation of Ser-6 alone can prevent excessive phosphorylation of RSG. However, the physiological importance of the autophosphorylation of Thr-21 remains elusive because the kinase activity of NtCDPK1 for RSG is already decreased by autophosphorylation of Ser-6 when Thr-21 is autophosphorylated. Gly-2 and Cys-4 in the variable N-terminal domain of NtCDPK1 are potential myristoylation and palmitoylation sites, respectively, that may associate the protein with the plasma membrane [17]. Ser-6 and Thr-21 are located close to these acylation sites, and GA treatment induces autophosphorylation of both Ser-6 and Thr-21. Therefore, GA-induced autophosphorylation of Ser-6 and Thr-21 would be assumed to cause the dissociation of NtCDPK1 from the plasma membrane. However, autophosphorylation does not actually affect the intracellular localization of NtCDPK1 [17,29]. We previously found that autophosphorylation decreases the phosphorylation efficiency of RSG, but increases that of myelin basic protein [29]. Although myelin basic protein was merely used as a substrate for activity measurement, this finding raises the possibility that autophosphorylated NtCDPK1 has physiological substrates other than RSG in plants, and that the substrate preference of NtCDPK1 may be altered by autophosphorylation. Autophosphorylation of Thr-21 could therefore be involved in the regulation of unknown physiological substrates. Alternatively, autophosphorylation of Thr-21 via an intramolecular mechanism might be required in cells where NtCDPK1 cannot form a homodimer, and/or where intermolecular autophosphorylation cannot occur due to low expression levels of NtCDPK1. Thus, autophosphorylation of Thr-21 might play a supportive role to that of Ser-6.

Cells generate transient increases in [Ca^2+^]_cyt_ that vary in amplitude, frequency, and duration in response to diverse environmental stimuli [2,41]. Different CDPKs evolved with divergent EF-hands of CaM-like domains with different Ca^2+^-binding affinities, which appears to enable different CDPKs to sense different Ca^2+^ thresholds [11]. Meanwhile, much is still unknown about how the duration of increased [Ca^2+^]_cyt_ affects the function of CDPKs. We found that autophosphorylation of Thr-21 requires a significantly longer period of time than that of Ser-6 (Fig 1), which might suggest that an increase in [Ca^2+^]_cyt_ for a short duration leads to autophosphorylation of Ser-6 alone, whereas a longer-lasting increase results in autophosphorylation of both Ser-6 and Thr-21 in plant cells. In conclusion, our findings suggest the possibility that short- and long-duration increases in [Ca^2+^]_cyt_ cause different autophosphorylation events in CDPKs.

## Supporting information

S1 Table Primer sequences used in this study. (PDF)
