# Supplementary material for: Autophosphorylation of Ser-6 via an intermolecular mechanism is important for the rapid reduction of NtCDPK1 kinase activity for substrate RSG
Source: PLoS One. 2018 Apr 23;13(4):e0196357. doi: 10.1371/journal.pone.0196357 (PMC5912773; doi:10.1371/journal.pone.0196357)
Supplement: S1 Table — (PDF) [file pone.0196357.s001.pdf]

S1 Table. Primer sequences used in this study.

**For protein expression vector**		
GST–NtCDPK1 S6A/D219N	5’-GGATCTGGTTCCGCGTGGAGCCATGGGTGGTTGTTTTGCCA AGAAG-3’	5’-CTCTGGCTTAAGATTCCTATGCATAACACC-3’
	5’-GGTGTTATGCATAGGAATCTTAAGCCAGAG-3’	5’-CACGATGCGGCCGCTCGAGCTAGAAAAGCTTCGGCTGTGG-3’
GST–NtCDPK1 T21A/D219N	5’-GGATCTGGTTCCGCGTGGAGCCATGGGTGGTTGTTTTAGCA AGAAG-3’	5’-CTCTGGCTTAAGATTCCTATGCATAACACC-3’
	5’-GGTATAGGGCAGCAAGAAGAAATGC-3’	5’-GCATTTCTTCTTGCTGCCCTATACC-3’
	5’-GGTGTTATGCATAGGAATCTTAAGCCAGAG-3’	5’-CACGATGCGGCCGCTCGAGCTAGAAAAGCTTCGGCTGTGG-3’
